# *Giardia duodenalis* in a clinically healthy population of captive zoo chimpanzees: Rapid antigen testing, diagnostic real-time PCR and faecal microbiota profiling

**DOI:** 10.1016/j.ijppaw.2022.03.007

**Published:** 2022-03-16

**Authors:** Christiana M. Willenborg, Barbora Červená, Paul Thompson, Eva Rosario, Craig Ruaux, Larry Vogelnest, Jan Šlapeta

**Affiliations:** a Sydney School of Veterinary Science, Faculty of Science, University of Sydney, New South Wales, 2006, Australia; b Department of Pathology and Parasitology, University of Veterinary Sciences Brno, Palackého třída 1946/1, 612 42, Brno, Czech Republic; c Institute of Vertebrate Biology, Czech Academy of Sciences, Květná 8, 603 65, Brno, Czech Republic; d Taronga Wildlife Hospital, Taronga Conservation Society Australia, Bradleys Head Road, Mosman, New South Wales, 2088, Australia

**Keywords:** Giardiasis, Diagnostics, Zoonosis, Zoo animals, Parasite, Commensal, Microbiome

## Abstract

*Giardia duodenalis* is one of the most common intestinal parasites of humans, with a worldwide distribution. *Giardia duodenalis* has been reported in both wild and captive populations of non-human primates, namely chimpanzees. In this study we investigated an entire troop of clinically healthy chimpanzees (n = 21) for the presence of *G. duodenalis* and its association with faecal microbiota profile. Faecal samples (n = 26) were collected from the chimpanzee exhibit from a zoo in Sydney, Australia. Diagnosis of *G. duodenalis* was made using a Rapid Antigen Test (RAT) as a point-of-care-test and compared to a reference standard real-time PCR test. Approximately half of the chimpanzee faecal samples tested positive for *G. duodenalis* by both RAT (13/26, 50%) and real-time PCR (14/26, 53.85%). The RAT sensitivity was 85.7% (95% CI: 63.8%–96%) and specificity was 91.7% (95% CI: 68.3%–99%) when compared to the in-house real-time PCR. Genotyping of the samples revealed the presence of zoonotic assemblage B. Microscopic analysis revealed the presence of *Troglodytella* spp. (14/26), *Balantioides* sp. (syn. *Balantidium* sp.) (8/26) as well as *Entamoeba* spp. (3/26). Microbiota profile based on 16S rRNA gene sequencing revealed that the community was significantly different between *G. duodenalis* positive and negative samples if RAT results were taken into an account, but not real-time PCR diagnostics results. Proteobacteria and Chloroflexi were the significant features in the dataset that separated *G. duodenalis* positive and negative samples using LEfSe analysis. Being able to rapidly test for *G. duodenalis* in captive populations of primates assists in point-of-care diagnostics and may better identify animals with subclinical disease. Under the investigated conditions of the zoo setting, however, presence of *G. duodenalis* either detected by RAT or real-time PCR was not associated with clinically apparent disease in captive chimpanzees.

## Highlights

• Whole troop investigation of healthy captive chimpanzees for *Giardia duodenalis*.
• Whole chimpanzee troop faecal microbiota profiled.
• Diagnosing *G. duodenalis* with Rapid Antigen Test (RAT) as a point-of-care-test.
• Comparison of RAT and reference real-time PCR test.
• Presence of *G. duodenalis* assemblage B.

## Graphical abstract

**Figure:**
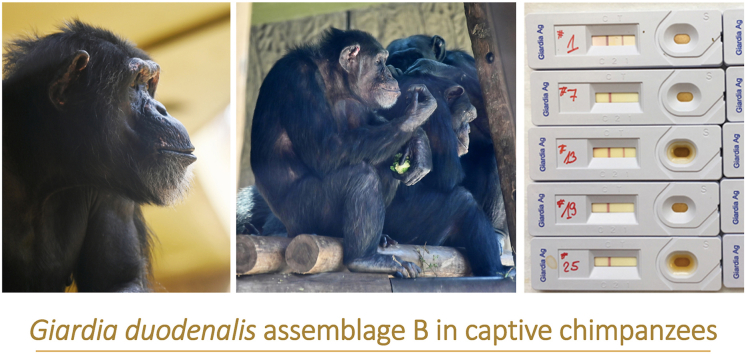

## 1 Introduction

Chimpanzees (*Pan troglodytes*) are great apes of the family Hominidae, known for their genetic similarity to humans (Urbanik and Johnston, 2017). It has been estimated that there are only approximately 345–470,000 chimpanzees left in the wild (Vigilant, 2004; Prado-Martinez et al., 2013; Humle et al., 2016). Their population numbers have declined over the last 40–50 years and they have been listed as endangered by the International Union for Conservation of Nature (IUCN) (Prado-Martinez et al., 2013; Dunay et al., 2018). They are found in 21 countries across the African continent, with the declining population of wild chimpanzees attributed to threats such as poaching, illegal trade, habitat destruction and disease (Dunay et al., 2018). About 1,600 chimpanzees are kept in approximately 650 facilities including zoos, aquariums, research and government organisations, and universities all over the world with 48 individuals held in four zoos across Australia (www.species360.org). The genetic similarity between chimpanzees and humans, together with expanding interactions between humans and chimpanzees due to eco-tourism, creates opportunities for pathogen transmission, including parasites (Muehlenbein, 2005; Dunay et al., 2018; Lonsdorf et al., 2021).

Parasite infections are quite common in nature and there is much discussion about the importance of such infections in wild populations of non-human primates, particularly chimpanzees (Ashford et al., 2000; Gillespie and Chapman, 2008; Frias et al., 2021; Mason et al., 2022; Petrželková et al., 2022). Field studies have been conducted on wild populations of chimpanzees across Africa and it has been shown that parasite populations vary across different groups of chimpanzees (Ashford et al., 2000; Muehlenbein, 2005; Gillespie and Chapman, 2008; Drakulovski et al., 2014). From opportunistic sampling of faecal samples in the wild at least 17 different taxa of parasites have been found, including helminths and protozoa. Some of the more common parasites isolated are *Troglodytella* sp., *Strongyloides* spp. and *Entamoeba* sp. (Muehlenbein, 2005; Drakulovski et al., 2014). While only found rarely (Gillespie et al., 2009; Sá et al., 2013; McLennan et al., 2017), the protozoan *Giardia duodenalis* (syn. *Giardia intestinalis, Giardia lamblia*) was detected in both chimpanzees and sympatric colobus monkey populations (Ashford et al., 2000; Gillespie and Chapman, 2008). Due to its zoonotic potential, occurrence of this protozoan is of public health concern as it could be transmitted to humans who share the environment with the chimpanzees or to those who look after captive chimpanzees.

*Giardia duodenalis* is known as a major cause of gastrointestinal disease in humans and other animals including livestock, and while it has been found in populations of wild chimpanzees, few studies have been done on the prevalence of this pathogen in captive chimpanzees (Lim et al., 2008; Beck et al.; Feng and Xiao, 2011; Debenham et al., 2015; Dunay et al., 2018). Six different studies have looked at intestinal parasites among captive animals including non-human primates housed in both zoos and rehabilitation centres across the world (Levecke et al., 2007; Lim et al., 2008; Beck et al., 2011; Berrilli et al., 2011; Martínez-Díaz et al., 2011; Debenham et al., 2015). Of these studies, five looked for *G. duodenalis* specifically and found varying results. One study did not detect *G. duodenalis* (Lim et al., 2008), another study found 70% (14/20 samples) prevalence of *G. duodenalis* in 10 different species of primates, with only one sample coming from a chimpanzee (Martínez-Díaz et al., 2011), the third study detected *G. duodenalis* in 39% (19/49 samples) of the chimpanzee samples collected from one site and zero from another site (Levecke et al., 2007), a fourth study found *Giardia* in five of twelve tested primate species, including chimpanzees (Beck et al., 2011) and the last study detected *G. duodenalis* in only 4.5% (7/154) faecal samples corresponding to 5.5% of individuals (5/90) in a Congolese chimpanzee sanctuary, while none of the chimpanzees (n = 10) in a Norwegian zoo tested positive (Debenham et al., 2015). These results were mainly obtained from individual samples, however, in some cases, pooled samples were all that was available for analysis due to the housing facilities of the animals (Levecke et al., 2007; Berrilli et al., 2011).

Although giardiasis, the disease caused by *G. duodenalis*, is commonly associated with anorexia, diarrhoea or vomiting in both humans and animal hosts the infection with *G. duodenalis* is commonly asymptomatic (Deplazes et al., 2016; Kváč and McEvoy, 2018; Fekete et al., 2020). Recent studies have shown an effect of *Giardia* infection on gastrointestinal microbiota with both positive and negative effects in humans (reviewed in Fekete et al., 2020), various animal species, including mice (Barash et al., 2017), dogs (Šlapeta et al., 2015; Berry et al., 2020; Boucard et al., 2021), cats (Šlapeta et al., 2015) and gold howler monkeys (Kuthyar et al., 2021). Alteration of the gut microbial community results in dysbiosis and subsequently may lead to development of gastrointestinal disease (Kriss et al., 2018).

Considering the parasite's zoonotic potential, it is important to be able to detect and further characterize *G. duodenalis* in clinical samples as well as determine its prevalence when chimpanzee hosts and humans are in close contact, whether that be as guests visiting the captive facilities or the people taking care of them such as zookeepers. Transmission of *G. duodenalis* from apes to humans and vice versa can occur via faecal-oral transmission and direct contact (Feng and Xiao, 2011; Kooriyama et al., 2013; Keita et al., 2014). Transmission of *G. duodenalis* occurs mainly via the faecal-oral route or through contaminated water sources, and while there is usually little chance for contamination of human water sources by *Giardia*-positive faeces from captive chimpanzees, there is still potential for transmission of this parasite through day-to-day contact with the keepers. As a result, continuous health monitoring of the captive chimpanzee population and of the keepers is important to reduce the risk of the disease spread (Berrilli et al., 2011; Kooriyama et al., 2013; Keita et al., 2014; Dunay et al., 2018).

The aim of this study was to investigate a complete troop (collective noun for apes) of captive chimpanzees housed at the Taronga Zoo, Sydney and determine the prevalence of different parasites occurring within the group. Specifically, we investigated the occurrence of *G. duodenalis* and its association with any distinct microbiota profile that could suggest that asymptomatic carriers could be suffering from dysbiosis. We further assessed the success of a point-of-care test (Rapid Antigen Test, RAT) for diagnosing *G. duodenalis* when compared to the reference standard real-time PCR test on faecal samples. The positive samples were genetically characterised to determine the zoonotic potential of *G. duodenalis* present within the chimpanzees at the zoo.

## 2 Materials and methods

### 2.1 Samples

The study was conducted at Taronga Zoo, Sydney during March 2019. The zoo was founded in 1916 and sits on 28 ha of land, and houses approximately 4,500 animals from almost 300 different species. The study was approved and agreed upon with the Taronga Conservation Society to collect opportunistic chimpanzee faecal samples for subsequent laboratory analysis (Taronga Opportunistic sample number R18L274). Veterinary records of the chimpanzees at Taronga Zoo were interrogated for clinical signs of gastrointestinal disturbance, such as diarrhoea and/or vomiting. The chimpanzees were housed in an exhibit with multiple sanctuaries. The main part of the exhibit was open air with climbing structures scattered throughout. There was a smaller exhibit enclosed with a mesh across the top and climbing structures. All chimpanzees have access to both indoor and outdoor dens and are given free range of their enclosures daily. The chimpanzees have several indoor dens with ledges and bedding material. At the time the study was conducted the chimpanzees had access to outside enclosures overnight. They usually build their night nests at dusk to sleep when its dark and then wake at dawn. They usually sleep inside but some will wake during the night and wander outside before coming back to bed.

A total of 26 faecal samples were collected from the chimpanzee enclosure at Taronga Zoo on the morning of 7 March 2019 (Sydney, Australia, Autumn). The outdoor enclosure is cleaned every morning at the same time before the chimpanzees are let outside into the enclosure for the day. Thus, the samples were collected opportunistically from the outdoor part of the exhibit during this regular morning clean up and kept individually in sampling vials. Faecal samples collected for the study were therefore no more than 24 h old. While we could not control the delay between defecation and sample collection, it is known that the time and temperature are not major factors when undertaking microbial profiling (Cunningham et al., 2020). It was not possible to assign the faecal samples to individual chimpanzees due to ethical, welfare and safety considerations. Individual faecal samples (C1 to C26) were immediately transported to the University of Sydney for processing and storage.

### 2.2 Rapid tests for Giardia duodenalis and coproscopic analyses

Before further processing all samples were tested for *G. duodenalis* using the Anigen Rapid Giardia AG Test Kit (RAT; BioNote, Seoul, South Korea) according to the manufacturer's instructions. Any sample where a faint line appeared in the ‘T’ position, along with the required ‘C’ line for control, within 10 min of the test window was recorded as positive.

All faecal samples were then processed using a Formalin-Ethyl Acetate Sedimentation technique as follows. Approximately 5g of fresh faeces was mixed with water and sieved into a 15 ml conical centrifuge tube and filled to the top. The tubes were centrifuged for 10 min at 1500g. The supernatant was poured off and 10 ml of 10% formalin was added to the sediment and mixed thoroughly with a wooden applicator stick. Four ml of ethyl acetate was added to the mixture and the tubes shaken vigorously to homogenize the faecal suspension. The tubes were again centrifuged for 10 min at 1500g. The plug that was formed was freed from the top of the sample and the supernatant was poured off. A few drops of 10% formalin were added to resuspend the sediment and the samples were stored at ambient temperature until further testing. For the microscopic examination of each of the 26 samples, one to three drops of the faecal sediment were transferred onto a slide and diluted with water to allow comfortable examination of the sample. A cover slip was added, and the slide was observed first under 100 × magnification and then 400 × using Olympus BX41 microscope (Olympus, Australia). The presence or absence of different intestinal parasites was recorded.

### 2.3 DNA isolation, detection, and sequencing

Approximately 2g of fresh faeces were aliquoted from the centre of each of the 26 faecal samples for DNA extraction and sequencing. A 150 mg sub-aliquot from each sample was homogenized and disrupted in a FastPrep-24 Homogenisation System equipped with QuickPrep Adapter (MP Biomedicals, Australia) at a speed setting of 6.0 m/s for 40 s prior to DNA isolation. The remaining aliquot of the fresh faeces was stored at −20 °C in case of need to repeat the analyses. Total DNA was then extracted from the 150 mg homogenized aliquot using the ISOLATE Faecal DNA Kit (BioLine, Australia) according to the manufacturer's instructions. The DNA isolation was processed in single batch along with negative control of PCR grade water to monitor for contamination. The extracted DNA was eluted into 100 μl aliquots of PCR-grade sterile water. All DNA aliquots were stored at −20 °C.

All extracted samples were tested for *G. duodenalis* using an in-house reference TaqMan real-time PCR as previously adopted (Meggiolaro et al., 2019). The TaqMan real-time PCR assay targets a 62-bp fragment of 18S rDNA of *G. duodenalis* (M54878) (Verweij et al., 2003). The TaqMan real-time PCR assay was shown to have high (>95%) sensitivity and specificity (Verweij et al., 2003; Uiterwijk et al., 2018).

A subset of five randomly selected real-time PCR positive samples was subjected to a conventional nested PCR assay in order to genotype the *Giardia* at 18S rDNA, triosephosphate isomerase 1 (TPI) gene and glutamate dehydrogenase (GDH) gene that are routinely used for *G. duodenalis* assemblage assignment (Feng and Xiao, 2011). The nested PCR assay for 18S rDNA used RH11 (5′-CAT CCG GTC GAT CCT GCC-3′), and RH4 (5′-AGT CGA ACC CTG ATT CTC CGC CCA GG-3′) for the first round and the nested GiarF (5′-GAC GCT CTC CCC AAG GAC-3′), and GiarR (5′-CTG CGT CAC GCT GCT CG-3′). primers producing ∼170 bp product. TPI gene was amplified by AL3543 (5′-AAA TIA TGC CTG CTC GTC G-3′) and AL3546 (5′-CAA ACC TTI TCC GCA AAC C-3′) primers in the first round followed by AL3544 (5′-CCC TTC ATC GGI GGT AAC TT-3′) and AL3545 (5′-GTG GCC ACC ACI CCC GTG CC-3′) primers producing a final ∼530 bp product. For the GDH gene we used two sets of primers: (i) primers that produce a ‘long’ fragment (∼530 bp) with Gdh1 (5′-TTC CGT RTY CAG TAC AAC TC-3′)/Gdh2 (5′-ACC TCG TTC TGR GTG GCG CA-3′) followed by Gdh3 (5′-ATG ACY GAG CTY CAG AGG CAC GT-3′)/Gdh4 (5′-GTG GCG CAR GGC ATG ATG CA-3′) and (ii) primers that produce a ‘short’ fragment (∼430 bp) with GDHeF (5′-TCA ACG TYA AYC GYG GYT TCC GT-3′)/GDHiR (5′-GTT RTC CTT GCA CAT CTC C-3′) followed by GDHiF (5′-CAG TAC AAC TCY GCT CTC GG-3′)/GDHiR. The initial real-time PCR screening was performed using the MyTaq Red Mix (BioLine, Australia) in a final volume of 30 μl according to manufacturer's instructions and then run for 35 cycles in a T100 PCR cycler (BioRad, Australia) with annealing temperature set at 55 °C. The nested PCR reactions used 1 μl of primary PCR as template. PCR products were separated on a 1.5% agarose gel to detect the single DNA band of expected size (Meggiolaro et al., 2019). PCR products were purified and bidirectionally sequenced using amplification primers at Macrogen Inc. (Seoul, Korea). Sequences were assembled and aligned with reference sequences representing *G. duodenalis* assemblage A–H using CLC Main Workbench 22 (CLC bio, a QIAGEN Company, Denmark).

### 2.4 Statistical analyses of Giardia duodenalis tests

To compare the RAT to the *Giardia-*specific real-time PCR, sensitivity, specificity and kappa statistics were calculated using a 2 × 2 contingency table. Confidence intervals were calculated using the modified Wald Method.

### 2.5 Microbial profiling and microbiota analysis

DNA aliquots were analysed for microbial diversity profiling at the Australian Genome Research Facility (Brisbane, Australia). Sequencing of the V3–V4 region of the 16S rRNA gene was performed on Illumina MiSeq (300bp pair-end) using primers 341F (5′-CCT AYG GGR BGC ASC AG-3′) and 806R (5′-GGA CTA CNN GGG TAT CTA AT-3′). Paired-ends reads were assembled using PEAR (version 0.9.5). Primers were identified and trimmed. Trimmed sequences were processed using Quantitative Insights into Microbial Ecology (QIIME 1.8/1.9.1), USEARCH (version 8.0.1623) and UPARSE software. Sequences were quality filtered clustered followed by chimera filtered using “rdp_gold” database as reference. To obtain the number of reads in each Operational Taxonomic Unit (OTU), reads were mapped back to OTUs with a minimum identity of 97%. QIIME taxonomy was assigned using Greengenes database (Version 13_8, Aug 2013).

Data were imported into ‘MicrobiomeAnalyst’ (https://www.microbiomeanalyst.ca/), a web-based tool used to analyse the data (Chong et al., 2020). The dataset was first cleaned to increase validity of results, OTUs resembling plant matter and nonbacterial species were removed as well as OTUs that did not classify beyond the Bacteria domain. This process decreased the number of OTUs from 1,470 to 1,447. OTU tables, count data and metadata was then input into the program and proceeded to undergo filtering and normalisation to remove low quality features to improve validity of analysis. Low count filter was set to a minimum count of 10 and 20% prevalence in samples. Low variance filter based on the inter-quantile range removed samples below 5% (inter-quantile range). Data were then rarefied to the lowest sequence read number (∼28,000 reads) and transformed using relative log transformation. Initial exploration of rarefaction curves and stacked bar plots revealed C14 and C17 samples as outliers with highly skewed stacked bar plots and distinct rarefaction curves with reduced alfa diversity, and thus were removed from the final analysis. C14 and C17 samples might have been from animals that were recently treated with antibiotics that reduced intestinal microbiota abundance due unintentional extinguishing of beneficial commensal microbes; antibiotics lead to temporal dysbiosis and overgrowth of undesirable bacterial groups which disruption intestinal homeostasis and function (Hasan and Yang, 2019). Metadata using the diagnostic tests (either RAT, real-time PCR and combination) for *G. duodenalis*, *Balantioides* sp. (syn. *Balantidium* sp.) and *Troglodytella* sp. were used to split the dataset. Alpha diversity profiling, beta diversity and linear discriminant analysis effect size (LEfSe) were assessed on every taxonomic level. Alpha diversity used diversity Shannon's index and observed number of OTUs and was compared using T-test. Beta diversity used ordination method based on Bray-Curtis dissimilarity matrix to calculate principal component analysis (PCoA) plots. Bray Curtis dissimilarity matrix was used for analysis of similarities (ANOSIM). LEfSe utilises the Kruskal-Wallis rank sum test to determine and compare significant differences in abundance among bacterial groups. Results were determined significant if the p-value < 0.05.

### 2.6 Data accessibility

The sequence data were deposited at SRA NCBI under BioProject ID: PRJNA796692. The nucleotide sequence data generated in this study was deposited in GenBank (NCBI): OM302215-OM302220. Associated data are available from LabArchives: https://dx.doi.org/10.25833/j8my-g081.

## 3 Results

### 3.1 Health status of captive chimpanzees

**Fig. 1:**
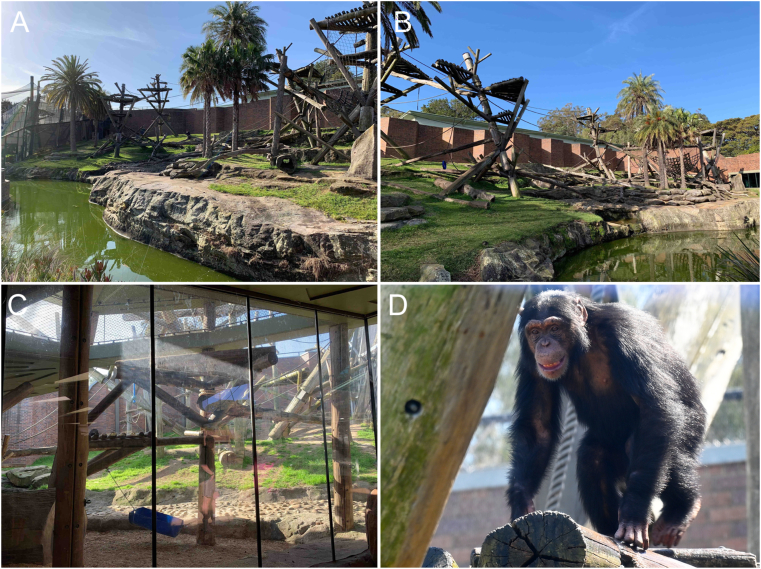
**Captive chimpanzees and their enclosure in Sydney, Australia.** (A) Main chimpanzee open air exhibit with multiple climbing structures. (B) View from the other direction showing entry to the indoor area at the end of the exhibit. (C) smaller exhibit with mesh covering and more climbing and sleeping structures. (D) Members of the chimpanzee troop at the Taronga Zoo.

At the time of sampling, the chimpanzee troop at Taronga Zoo included 21 chimpanzees ranging in age from one to 58 years, with 12 females and 9 males (Table 1; Fig. 1). All chimpanzees in the troop appeared to be in good health and did not show any signs of gastrointestinal problems during the period of 3 months prior and 3 months post faecal sampling. During the study period, five chimpanzees received medications for reasons other than gastrointestinal issues, and two of them had transient diarrhoea noted in the veterinary records because of antibiotic use (Table 2). All samples (n = 26) collected were formed and non-diarrhoeic faeces.

**Table 1:** Demographics of the chimpanzee troop at Taronga Zoo, Sydney from which faecal samples were collected for analysis.

Age	Male	Female	Total
Less than 5 years	2	1	3
5 years and older	7	11	18
Total	9	12	21

The number corresponds to the time of sampling in March 2019.

**Table 2:** Chimpanzee medication use during the study period.

Chimpanzee	Medication (antibiotics*)	Reason	Diarrhoea present
Chimpanzee 1	Meloxicam, *Amoxicillin/clavulanic acid	Suspect bite wound	Yes
Chimpanzee 2	Meloxicam and *Amoxicillin/clavulanic acid	Foot injury	No
Chimpanzee 3	Meloxicam	Wrist lameness	No
Chimpanzee 4	Paracetamol and *Amoxicillin	Amputated digit	No
Chimpanzee 5	*Amoxicillin, Meloxicam and *Amoxicillin/clavulanic acid	Bite wounds	Yes

### 3.2 High prevalence of Giardia duodenalis in captive chimpanzees

**Fig. 2:**
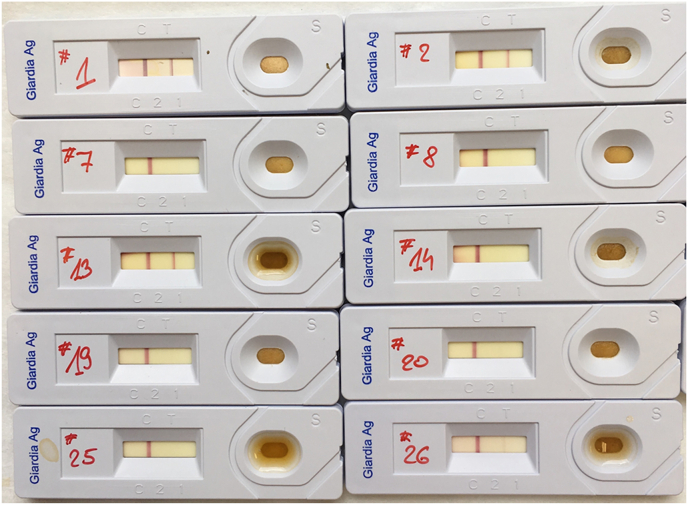
| **Results of *Giardia duodenalis* rapid antigen test applied on faecal samples from chimpanzees.** A positive result for the *Giardia duodenalis* rapid antigen test (RAT, Anigen Rapid Giardia AG Test Kit) is represented by the line in the ‘T’ position in the window along with the positive control line in the ‘C’ position.

The faecal samples were analysed using a *Giardia-*specific real-time PCR, Anigen Rapid Giardia AG Test Kit (RAT) and Formalin-Ethyl Acetate Sedimentation Concentration for other parasites (Table 3; Supplementary Data 1). The proportion of *G. duodenalis* positive faecal samples was 14/26 (53.9%; 95% CI: 35.5%–71%) for the real-time PCR and 13/26 (50%; 95% CI: 32%–67.9%) for the RAT. All RATs were valid, with the positive control line appearing in all tests conducted (Fig. 2). The real-time PCR results returned C_t_ values below the cut-off of 35 (19.01–30.28) cycles. Between the two tests, there was a substantial agreement, Kappa = 0.769. Two samples tested negative by RAT, but positive by real-time PCR and one sample tested positive by RAT while real-time PCR did not show any *Giardia* DNA.

**Table 3:** Parasites detected in faecal samples from captive chimpanzees at Taronga Zoo, Australia.

Sample	*Giardia duodenalis*		*Troglodytella* sp.	*Balantioides* sp.	*Entamoeba* sp.
	RAT	real-time PCR			
C1	+	+	+		+
C2	+	+			
C3		+	+		+
C4	+	+			
C5					
C6			+	+	
C7*			+	+	
C8			+	+	
C9			+	+	
C10			+		
C11			+		+
C12	+	+	+		
C13	+	+	+		
C14					
C15	+	+	+	+	
C16	+	+			
C17	+	+		+	
C18	+	+			
C19			+		
C20		+	+		
C21	+	+			
C22	+	+			
C23					
C24	+	+		+	
C25				+	
C26	+		+		

Note: * - additional pseudoparasite eggs (tapeworms) detected; RAT - Anigen Rapid Giardia AG Test Kit (BioNote, Seoul, South Korea); real-time PCR - TaqMan real-time PCR assay targets a 62-bp fragment of 18S rDNA of *G. duodenalis* (M54878) (Verweij et al., 2003).

Sensitivity of the RAT test was 85.7% (95% CI: 63.8%–96%) and specificity was 91.7% (95% CI: 68.3%–99%) when compared to the in-house real-time PCR. The proportion of other parasite positive faecal samples was 23/26 (88.5%; 95% CI: 70.2–96.8%). *Giardia* cysts were not observed during coproscopic examination, however, other parasites detected via microscopic analysis included *Troglodytella* sp. (14/26), *Balantioides* sp. (syn. *Balantidium* sp.) (8/26), *Entamoeba* spp. (3/26) and a potential cestode pseudoparasite (tapeworm) (1/26).

### 3.3 Presence of G. duodenalis assemblage B in captive chimpanzees

Of the 14 *G. duodenalis* real-time PCR positive faecal samples, five (36%, 5/14) were randomly selected and processed for further molecular analysis to determine the *G. duodenalis* assemblage. The SSU rDNA amplicon (140bp) from chimpanzee faecal sample C1 and C4 revealed 100% identity to *G. duodenalis* assemblage B reference SSU rDNA sequences (GenBank Accession numbers U09491 and AF199447). The TPI gene amplicon (489bp) from chimpanzee sample C1 and C2 was 100% identical to a reference TPI gene sequence from *G. duodenalis* assemblage B (GenBank Accession number EF688023). Using two independent GDH gene amplicons (393bp and 497bp) from chimpanzee sample C1, C3, C4 and C15, we confirmed 100% identity with reference *G. duodenalis* assemblage B GDH gene sequence (GenBank Accession number AY178738).

### 3.4 Microbiota profile between G. duodenalis positive and negative faecal samples is dependent on the Giardia-diagnostic tool in captive apparently healthy chimpanzees

**Fig. 3:**
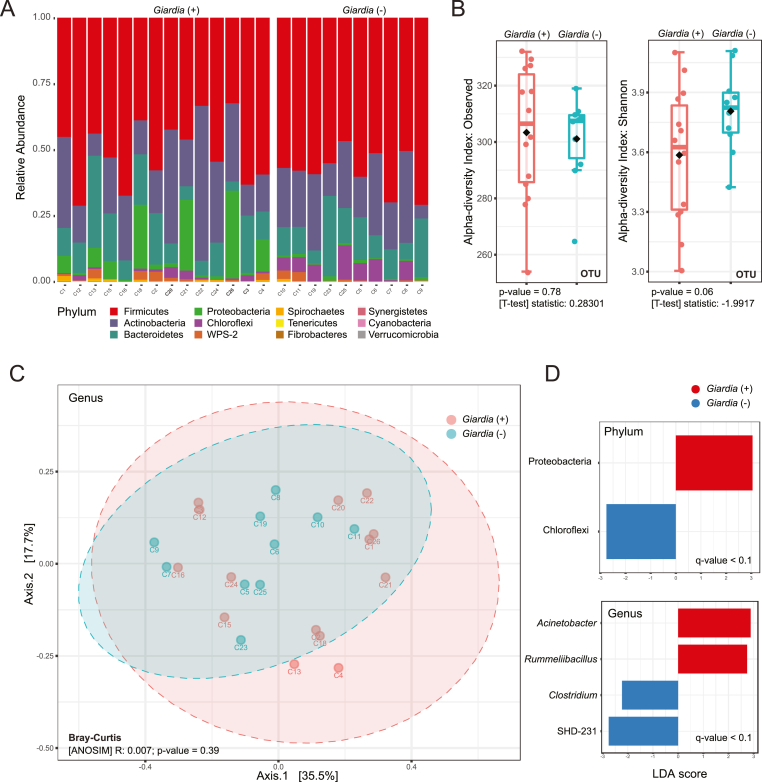
| **Faecal bacterial community profile of captive chimpanzees infected with *Giardia duodenalis* as detected by rapid antigen test and real-time PCR combined.** (A) Relative abundance of colour coded bacterial phyla separated based on presence (+) or absence (−) of *Giardia*. The sample identity is located at the bottom of the graph. (B) Alpha diversity based on observed OTU and Shannon's index plotted as box plot and evaluated using t-tests. (C) Principal coordinates analysis (PCoA) 2D plot using first two principal components from Bray-Curtis dissimilarity matrix at the genus taxonomic levels. The clustering between *Giardia* positive (+) and negative (−) samples was tested using ANOSIM. (D) Linear discriminant analysis effect size (LEfSe) used plot of significant factors discriminating *G. duodenalis* positive from negative sample. (For interpretation of the references to colour in this figure legend, the reader is referred to the Web version of this article.)

We obtained 16S rDNA microbial profiles for 14 *G. duodenalis* positive samples and 10 *G. duodenalis* negative faecal samples, based on pooled RAT and real-time PCR results. The sequence reads per sample average was 68,060 (28,599–104,440). After taxonomic assignment, filtering and removal of low abundance OTUs, the final dataset included 368 OTUs. The microbiota in the dataset rarefied to the minimum library size (28,599 reads) was dominated by Firmicutes, Actinobacteria, and Bacteroides (Fig. 3A). Visually, the *G. duodenalis* positive samples appeared to have inverse proportions of Proteobacteria and Chloroflexi (Fig. 3A). Alpha diversity at the OTU level, measuring richness and evenness within samples, demonstrated no significant difference between *G. duodenalis* positive and negative samples (Fig. 3B). We then compared beta diversity between the *G. duodenalis* positive samples and *G. duodenalis* negative faecal samples (Fig. 3C). In PCoA on genus level, the first axis captured >35% diversity but there was no clear division between the positive and negative samples, further confirmed by ANOSIM analysis (R = 0.007; p-value = 0.39). To inquir into *G. duodenalis* load (C_t_-value; Supplementary Table S1) we split the samples into those with high (C_t_-value = 19.01–20.88) and low (C_t_-value = 21.98–30.28) load but could not demonstrate any significant separation between the *G. duodenalis* high, low and negative samples (ANOSIM: R = −0.043; p-value = 0.70). Similarly, there was no significant separation at any taxonomic level using ANOSIM analysis (p-value>0.05). The initial observation revolving around Proteobacteria and Chloroflexi was confirmed using LEfSe analysis, demonstrating significantly more abundant Proteobacteria in *G. duodenalis* positive samples while Chloroflexi were significantly more abundant in *G. duodenalis* negative samples (Fig. 3D). At the genus level, LEfSe demonstrated four genera as the significant features between positive and negative samples (Fig. 3D).

**Fig. 4:**
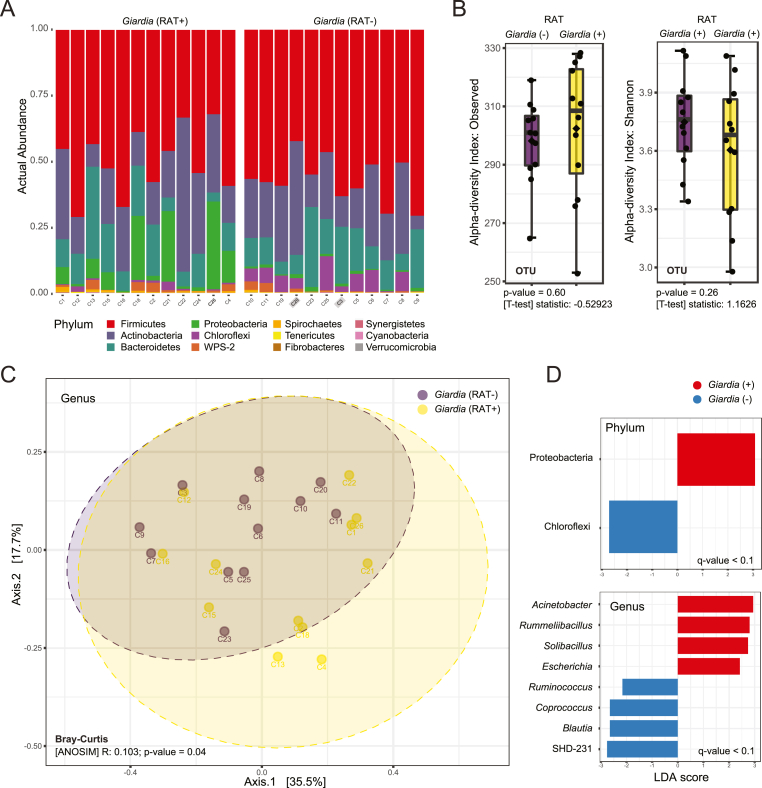
| **Faecal bacterial community profile of captive chimpanzees infected with *Giardia duodenalis* detected by rapid antigen test.** (A) Relative abundance of colour coded bacterial phyla separated based on presence (+) or absence (−) of *Giardia* using rapid antigen test (RAT). The sample identity is located at the bottom of the graph with two labels (C20, C3) shaded indicating samples that were found as *Giardia* positive by real-time PCR. (B) Alpha diversity based on observed OTU and Shannon's index plotted as box plot and evaluated using t-tests. (C) Principal coordinates analysis (PCoA) 2D plot using first two principal components from Bray-Curtis dissimilarity matrix at the genus taxonomic levels. The clustering between *Giardia* positive (RAT+) and negative (RAT-) samples was tested using ANOSIM. (D) Linear discriminant analysis effect size (LEfSe) used plot of significant factors discriminating *G. duodenalis* positive from negative sample. (For interpretation of the references to colour in this figure legend, the reader is referred to the Web version of this article.)

We then repeated the analysis with only the *G. duodenalis* RAT classification as either positive or negative sample (Fig. 4). Two samples, C3 and C20, were RAT negative but real-time PCR positive for *G. duodenalis* antigen and DNA, respectively (Fig. 3, Fig. 4). The inverse proportions of Proteobacteria and Chloroflexi was further pronounced on visual inspection, because both re-categorized samples had low proportion of Proteobacteria (Fig. 4A). There was no significant difference in alpha diversity at the OTU level between *G. duodenalis* positive and negative samples (Fig. 4B). At the genus level, there was a significant division using ANOSIM analysis (R: 0.103; p-value = 0.04) (Fig. 4C). Similarly, there was a significant separation at all taxonomic levels using ANOSIM analysis (p-value<0.05). Proteobacteria and Chloroflexi were the significant features in the dataset that separated the RAT positive and negative samples using LEfSe analysis in addition to eight genera (Fig. 4D). At any taxonomic level there was no significant division using ANOSIM analysis for either *Troglodytella* sp. or *Balantioides* sp. (syn. *Balantidium* sp.) positive/negative samples (p-value>0.05).

## 4 Discussion

The current study indicates that at least half of a captive chimpanzee population was infected with *G. duodenalis*. The presence of *G. duodenalis* has been demonstrated previously in captive chimpanzees (Levecke et al., 2007; Beck et al., 2011; Berrilli et al., 2011; Martínez-Díaz et al., 2011; Debenham et al., 2015). To the best of our knowledge this is the first study that has assessed the prevalence of *G. duodenalis* in an entire troop of 21 captive chimpanzees. A single mass collection provided in depth information about the presence of different parasites within the chimpanzee troop. It is unlikely that fewer than the whole troop produced all 26 individual samples collected, so we can be confident that faecal samples analysed represent the whole captive troop at Taronga Zoo, Sydney. However, the true number of infected animals may be much higher, because *G. duodenalis* is often shed intermittently (Danciger and Lopez, 1975). Health records of this group showed that no signs corresponding to gastrointestinal pathologies occurred within the group at the time of sampling. Absence of such clinical signs could indicate that either this parasite is a part of normal gut flora of this chimpanzee troop, or they represent an asymptomatic infection as is often observed in animals (Feng and Xiao, 2011).

The presence of *G. duodenalis* assemblage B in the chimpanzee troop indicates horizontal transmission among the animals in the troop (Debenham et al., 2015). The reason why the infection is so prevalent in these chimpanzees and the source of *G. duodenalis* is currently unknown. It is possible that *G. duodenalis* was introduced with a single chimpanzee that served as the source for the troop. But as human *G. duodenalis* infections are connected to contaminated water sources (Sprong et al., 2009; Ryan and Cacciò, 2013; Heyworth, 2016). There is a possibility that the vehicle of *G. duodenalis* contamination at Taronga Zoo could be water available to the chimpanzees or, more probably, water from a moat separating the enclosure from the visitor area. Chimpanzees do not swim however they have been observed using sticks to retrieve things from the moat to play with and have been seen interacting with various waterfowl that intermittently use the moat water.

In the present study, we used a point-of-care RAT for detecting *G. duodenalis* infection and compared the results to those of the reference real-time PCR in captive chimpanzees. Presentation of dogs and cats to veterinary clinics with diarrhoea associated with *Giardia* infection is quite common and the use of the rapid in-clinic tests is preferred for quick diagnosis (Barbecho et al., 2018; Meggiolaro et al., 2019). Previously, in-clinic tests for detection of *Giardia* in canine faeces available at small veterinary clinics were compared to a reference laboratory test, this study showed that RATs represent a valuable diagnostic tool (Barbecho et al., 2018). We confirmed that RATs are suitable for detection of *G. duodenalis* in chimpanzees. Our results and those of the previous studies indicate that *G*. *duodenalis* is common in captive chimpanzees (Levecke et al., 2007; Beck et al., 2011; Berrilli et al., 2011; Martínez-Díaz et al., 2011; Debenham et al., 2015; Zhong et al., 2017) but neither of the previous studies addressed the potential pathogenicity of *G. duodenalis* to chimpanzee hosts.

In this study we noted a correlation between *G. duodenalis* infection detected using RAT and microbial disturbance through beta diversity. This finding, however, was not supported if DNA based diagnostic real-time PCR was used, or separating the samples based on burden based on real-time PCR C_t_-values. Such results add to the conundrum surrounding the asymptomatic *G. duodenalis* infections and complex pathogenesis (Robertson et al., 2010; Tysnes et al., 2014; Fekete et al., 2020). By design our investigation lacks information on chimpanzee identities, and thus individual host factors contributing to the *G. duodenalis* infection. This limitation, however, does not prevent us from indicating possible associations between *G. duodenalis* and the microbiota. The microbiota associated with *G. duodenalis* has previously been associated with altered proportions of bacterial groups, it has been proposed that the degree of this alteration influences whether the infection remains asymptomatic or manifests as clinical giardiasis (Barash et al., 2017; Kriss et al., 2018; Fekete et al., 2020). The resulting dysbiosis is strongly correlated to many intestinal disorders, such as irritable bowel disease (IBD) and diarrhoea (Wohlgemuth et al., 2009; Loh and Blaut, 2012; Pilla and Suchodolski, 2019; Fekete et al., 2020). In dogs, Pila and Suchodolski (2020) provided evidence for a decreased microbial diversity, proceeding to a rise of *Clostridium* and reduction of *Blautia* spp. and *Ruminococcus* spp. which produce short-chain fatty acids, compounds vital for proper gastrointestinal function. In our study, Proteobacteria was found to be the phylum differentiating *G. duodenalis* positive from *G. duodenalis* negative chimpanzee faecal samples. Proteobacteria are normally found in healthy animals, however, dysbiosis and gut inflammation is linked to high proportions of this phylum (Moon et al., 2018). Our results align with previous studies of canine and murine faecal microbiota in *Giardia* infected animals which also showed increased proportions of Proteobacteria (Šlapeta et al., 2015; Barash et al., 2017). In contrast with those studies, Firmicutes have not shown decreased abundance in *Giardia* infected chimpanzees (Suchodolski et al., 2012; Šlapeta et al., 2015). Diet is known to be a major factor governing the overall microbiota in various hosts (Li et al., 2016; Singh et al., 2017; Hicks et al., 2018; Leeming et al., 2021) which may be of great significance in captive animals such as our study subjects. It is possible that non-optimal diet creates permissive gut conditions allowing *Giardia* to establish, which could further imply that presence of *G. duodenalis* is an indicator of non-healthy gut and microbiota disbalance.

The presence of *G. duodenalis* assemblage B in captive chimpanzees raises a question about its capacity to infect humans (Koster et al., 2021). Eight different assemblages (A-H) of *Giardia* spp. have been named (Feng and Xiao, 2011; Heyworth, 2016; Brynildsrud et al., 2018). Humans are only infected by assemblage A and B with one study showing most reported cases (86%) to be assemblage B (Feng and Xiao, 2011; Asher et al., 2016). Transmission between humans and animals is rare, however a human assemblage has previously been identified in cattle that lived near humans (Feng and Xiao, 2011; Asher et al., 2016). Similarly, free-ranging populations of non-human primates have been reported to be infected predominantly by assemblage B, along with other parasites, as a result of closely sharing a habitat with humans, the type of farming practices used, livestock movements and contamination with human faeces as they move through the land (Ashford et al., 2000; Gillespie et al., 2010; van Zijll Langhout et al., 2010; Ryan and Cacciò, 2013). Due to the naivety of the chimpanzee immune system, the increasing spread of human-borne disease through ecotourism has also been identified as attributing to the decline of wild chimpanzees and other non-human primate populations (Kooriyama et al., 2013; Dunay et al., 2018).

Other parasite taxa detected by the coproscopic examination of the faecal samples comprised protozoans of the genera *Troglodytella*, *Balantioides* (syn. *Balantidium*) and *Entamoeba*, all of them endosymbionts commonly detected in captive chimpanzees (Jirků Pomajbíková and Modrý, 2018; Jirků Pomajbíková and Vlčková, 2018; Profousová-Pšenková, 2018; Koster et al., 2021). Even though reported together with the parasites, entodiniomorphid ciliates (*Troglodytella* spp.) are commensals or mutualists contributing actively to the hindgut starch and fiber fermentation (Profousová et al., 2011). Interestingly, *Troglodytella* seems to be the only genus from the group occurring in captive chimpanzees and bonobos, and its abundance was shown to be positively correlated with the amount of starch in the diet (Pomajbíková et al., 2010; Petrželková et al., 2012). High starch diets led to an increase in quantities of *Balantioides coli*, an isotrochid ciliate known to cause clinical, occasionally lethal balantidiasis in captive chimpanzees (Kim et al., 1978; Schovancová et al., 2013). Finally, the genus *Entamoeba* comprises several species, mainly of which are commensals, but also some pathogens: *E. histolytica* and *E. nuttali* (Jirků Pomajbíková and Vlčková, 2018). Unfortunately, it is impossible to reliably determine *Entamoeba* species without using DNA methods (Kebede et al., 2004; Vlčková et al., 2018). No attempt was made to sequence the *Entamoeba* spp. occurring in the investigated chimpanzees, due to low detection rate within the troop. All the detected protozoans are transmitted by the orofecal route and therefore, the infection is likely maintained as such in the captive setting. As is the case with *Giardia* both entodiniomorphid and isotrichid ciliates seem to be greatly influenced by the diet composition with potential impact on the health in the case of *Balantioides*. Optimization of the diet in captive animals is therefore crucial for the overall wellbeing of the animals.

*Giardia duodenalis* infection in wild chimpanzees in undisturbed environments is rare, which is in contrast with captive animals or those living in fragmented habitats (Gillespie et al., 2009; Pomajbíková et al., 2010; Drakulovski et al., 2014; Debenham et al., 2015; Koster et al., 2021). An explanation could include the reverse-zoonotic ability of *G. duodenalis* or infection from domestic animals via waterborne routes (Gillespie et al., 2009; Debenham et al., 2017; Kuthyar et al., 2021). Studies comparing microbiota, *Giardia-*infection rate and diet in different captive chimpanzee troops are needed to determine if the diet contributes to intestinal microbial changes and aids *Giardia* colonisation, which would imply that the diet currently provided to the captive chimpanzees needs to be reformulated. Similarly, extension of studies such as Hicks et al. (2018) that consider presence/absence of parasites would improve our understanding of the degree that *G. duodenalis* in chimpanzees is a noteworthy pathogen or a harmless commensal.

## 5 Conclusions

Investigation of the entire troop of clinically healthy captive chimpanzees for the presence of *G. duodenalis* and its association with faecal microbiota profile adds to the conundrum of the role this parasite plays in the gut health in animals and humans. Repeated sampling, enumeration of *G. duodenalis* cysts and identification of the faecal samples to individuals could improve the understanding of the role of *G. duodenalis* within the troop. The Assemblage B of *G. duodenalis* is not causing clinically apparent disease in the investigated captive chimpanzees. Due to the single sampling event, we cannot exclude the possibility that all members of the troop are latently infected with *G. duodenalis*.

## Declaration of competing interest

The authors declare that they have no known competing financial interests or personal relationships that could have appeared to influence the work reported in this paper.
